# Viral UL8 Is Involved in the Antiviral Activity of Oleanolic Acid Against HSV-1 Infection

**DOI:** 10.3389/fmicb.2021.689607

**Published:** 2021-07-20

**Authors:** Tianhao Shan, Ju Ye, Jiaoyan Jia, Zhaoyang Wang, Yuzhou Jiang, Yiliang Wang, Yifei Wang, Kai Zheng, Zhe Ren

**Affiliations:** ^1^Institute of Biomedicine, College of Life Science and Technology, Jinan University, Guangzhou, China; ^2^Key Laboratory of Virology of Guangzhou, Jinan University, Guangzhou, China; ^3^Key Laboratory of Bioengineering Medicine of Guangdong Province, Jinan University, Guangzhou, China; ^4^Department of Cell Biology, College of Life Science and Technology, Jinan University, Guangzhou, China; ^5^Guangdong Provincial Biotechnology Drug and Engineering Technology Research Center, Guangzhou, China; ^6^National Engineering Research Center of Genetic Medicine, College of Life Science and Technology, Jinan University, Guangzhou, China; ^7^Key Laboratory of Plant Chemistry in Qinghai-Tibet Plateau, Qinghai University for Nationalities, Xining, China; ^8^School of Pharmaceutical Sciences, Health Science Center, Shenzhen University, Shenzhen, China

**Keywords:** herpes simplex virus type 1, Oleanolic acid, UL8, helicase-primase complex, zosteriform model

## Abstract

Herpes simplex virus type 1 (HSV-1) is highly prevalent in humans and can cause severe diseases, especially in immunocompromised adults and newborns, such as keratitis and herpes simplex encephalitis. At present, the clinical therapeutic drug against HSV-1 infection is acyclovir (ACV), and its extensive usage has led to the emergence of ACV-resistant strains. Therefore, it is urgent to explore novel therapeutic targets and anti-HSV-1 drugs. This study demonstrated that Oleanolic acid, a pentacyclic triterpenoid widely existing in natural product, had strong antiviral activity against both ACV-sensitive and -resistant HSV-1 strains in different cells. Mechanism studies showed that Oleanolic acid exerted its anti-HSV-1 activity in the immediate early stage of infection, which involved the dysregulation of viral UL8, a component of viral helicase-primase complex critical for viral replication. In addition, Oleanolic acid significantly ameliorated the skin lesions in an HSV-1 infection mediated zosteriform model. Together, our study suggested that Oleanolic acid could be a potential candidate for clinical therapy of HSV-1 infection-related diseases.

## Introduction

Herpes simplex virus type 1 (HSV-1) is an enveloped DNA virus belonging to *alphaherpesvirinae* subfamily (Midak-Siewirska et al., 2010). HSV-1 can cause severe diseases in immunocompromised adults and newborns, such as herpes simplex encephalitis and keratitis. Infection of HSV-1 also triggers oral and facial lesions (clinical manifestation as *Herpes labialis*), as well as skin lesions (*Herpes gladiatorum*) (Lafferty et al., 1987; Mertz et al., 1998; Kaye and Choudhary, 2006; Paz-Bailey et al., 2007; Farooq and Shukla, 2012). Notably, *H. gladiatorum* typically passes among people via direct skin-to-skin contact and the virus enter neurons to establish latency and to cause neurite damage. Accordingly, accumulating evidence has shown that neurodegenerative diseases, such as Alzheimer’s disease and Parkinson’s disease, are associated with HSV-1 infection (Marttila et al., 1981; Caggiu et al., 2016; Agostini et al., 2018). At present, the clinical drug against HSV-1 infection is acyclovir (ACV), which targets HSV-1 DNA polymerase to inhibit viral replication (Hassan et al., 2015). However, the widespread usage of ACV has led to the clinical emergence of ACV-resistant strains. Therefore, it is urgent to explore novel antiviral targets and develop new drugs with antiviral mechanisms different from ACV.

HSV-1 DNA replication requires the primase activity of the helicase-primase complex, which is a heterotrimer consisting of the protein products of viral *UL5*, *UL8*, and *UL52* genes (Crute et al., 1989; Boehmer and Lehman, 1997). The subcomponents of UL5 and UL52 process DNA-dependent ATPase, helicase and primase activities (Dodson and Lehman, 1991). Although the UL8 subunit has no known catalytic function, it interacts with other components and modulates the enzymatic functions of UL5/UL52 subcomplex. For instance, UL5/UL8/UL52 complex shows stronger activity than UL5/UL52 complex on single-strand DNA. The UL5/UL52 complex’s promoter activity is prevented by viral single-strand DNA binding protein ICP8, whereas UL8 specifically interacts with and releases ICP8 from single-strand DNA, enhancing the activity of UL5/UL8/UL52 complex (Carmichael and Weller, 1989; Tenney et al., 1994; Tanguy Le Gac et al., 1996; Falkenberg et al., 1997; Marsden et al., 1997). In addition, UL8 is necessary for the effective nuclear entry of the helicase-primase complex (Barnard et al., 1997). Therefore, the helicase-primase complex represents a promising alternative target for antiviral therapy, and inhibitors targeting UL5, UL52, or UL8 are potent drug candidates for the treatment of HSV-1 infection-related disease.

**FIGURE 1:**
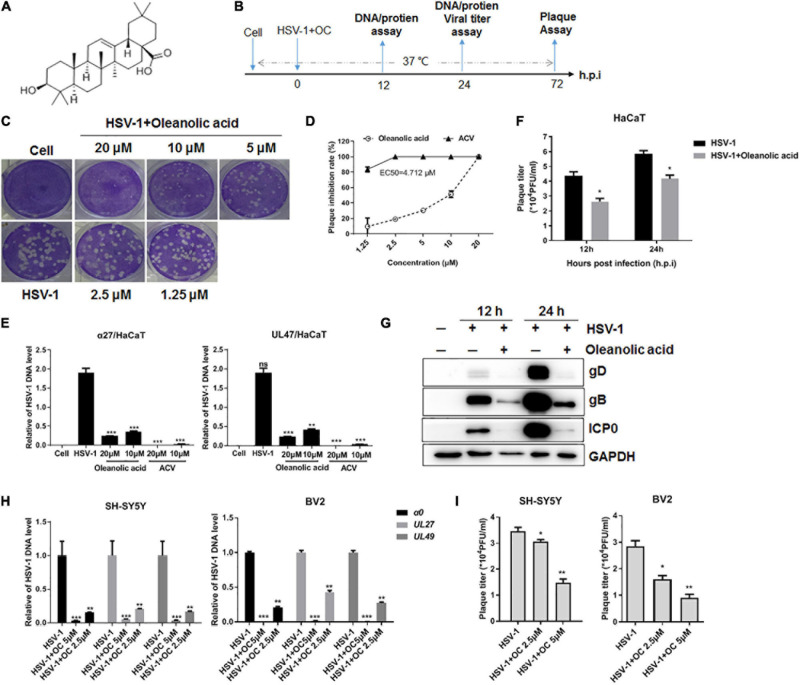
The anti-HSV-1 activity of Oleanolic acid. **(A)** Chemical structure of Oleanolic acid. **(B)** Simple diagram of the performed experiments. **(C,D)** Vero cells were infected with HSV-1 (MOI = 1) in the presence of Oleanolic acid or ACV at different concentrations for 72 h. The cells were then fixed and stained with crystal violet dye. The plaque numbers were counted to calculate the inhibition rate. Data are mean ± SD (*n* = 2). **(E)** HaCaT cells were treated with HSV-1 (MOI = 1) and Oleanolic acid for 24 h. Viral DNA was extracted and the DNA copy number of viral gene *α27* and *UL47* were determined by qRT-PCR. Data are mean ± SD (*n* = 3). **p < 0.01, ***p < 0.001. **(F,G)** HaCaT cells were treated with HSV-1 (MOI = 1) and Oleanolic acid for 12 or 24 h, respectively. Virus titration **(F)** and the expression levels of viral protein ICP0, gB, and gD **(G)** were detected. Data are mean ± SD (*n* = 3). *p < 0.5. **(H,I)** SH-SY5Y and BV2 cells were infected with HSV-1 (MOI = 1) in the presence or absence of Oleanolic acid (OC) at indicated concentrations for 24 h. Total viral DNA was extracted and the DNA copy numbers of viral genes α*0, UL27*, and *UL49* were determined by qRT-PCR **(H)**. The supernatant and cell pellets were collected to calculate virus titers **(I)**. Data are mean ± SD (*n* = 3). *p < 0.5, **p < 0.01, ***p < 0.001.

Many natural products have been demonstrated to have reasonable anti-HSV-1 activities, such as terpenes, polyphenols and alkaloids (Sagar et al., 2010). Oleanolic acid (Figure 1A) is a kind of pentacyclic triterpenoids widely existing in the whole plant kingdom. Oleanolic acid has multiple pharmacological activities, such as anti-tumor, anti-inflammation and liver protection (Oguro et al., 1998; Wang et al., 2006; Pollier and Goossens, 2012; Lee et al., 2013). In addition, few studies have revealed the anti-herpesvirus effects of Oleanolic acid. One study showed that Oleanolic acid could inhibit HSV-1 (EC_50_ 6.8 g/ml) and HSV-2 (EC_50_ 7.8 g/ml) via promoting the release of pro-inflammatory cytokine IL-6 and IL-12 (Mukherjee et al., 2013). In addition, 15 derivatives of Oleanolic acid triterpenoids also showed moderate anti-HSV-1 activities (Ikeda et al., 2005). However, the anti-HSV-1 mechanism of Oleanolic acid is still unclear.

Herein, we systematically evaluated the antiviral activity of Oleanolic acid against common HSV-1 and ACV-resistant HSV-1 strains *in vivo* and *in vitro.* We also explored its potential molecular mechanism, which involved the deregulation of viral UL8. These results suggest that Oleanolic acid has significant potential for the treatment of HSV-1 infection-induced skin lesions, such as *H. gladiatorum*.

## Materials and Methods

### Cells and Viruses

African green monkey kidney cell line (Vero cells) and Human immortalized keratinocyte cell line (HaCaT) were purchased from the American Type Culture Collection Center (ATCC). Vero, HaCaT, SH-SY5Y (ATCC CRL-2266), and BV2 (Cell Bank, Chinese Academy of Sciences) cells were cultured in Dulbecco’s modified Eagle’s medium (DMEM; 8118305, GIBCO/Thermo Fisher Scientific, United States) with 10% fetal bovine serum (FND500, ExCell Bio, Shanghai, China). HSV-1 F strain (ATCC, United States) preserved by the University of Hong Kong, was propagated in Vero cells and stored in the refrigerator at −80°C. HSV-1/Blue, a TK mutant derived from HSV-1 KOS strain and two clinical ACV-resistant HSV-1 strain, HSV-1/106 and HSV-1/153, were kind gifts from Prof Tao Peng (Guangzhou Institutes of Biomedicine and Health, Chinese Academy of Sciences, China). Green fluorescent protein (GFP)-tagged HSV-1 F strain (GFP-HSV-1) (GFP-tagged viral protein Us11) was obtained from the research group of Professor Yuan Li (Jinan University, China).

### Compounds, Antibodies, siRNAs, and Plasmids

Oleanolic acid was isolated from benzoin and was prepared by Shenzhen University. Oleanolic acid was dissolved in dimethyl sulphoxide with a concentration of 50 mM. Acyclovir (ACV) was purchased from Sigma-Aldrich (St. Louis, MO, United States). Antibodies, including anti-gB (ab6506), anti-ICP0 (ab6513), anti-gD (ab6507), anti-VP16 (ab11026), anti-ICP4 (ab6514), anti-ICP8 (ab20194), anti-ICP27 (ab53480), anti-ICP22 (ab6506), were purchased from Abcam (Cambridge, United Kingdom), anti-β-actin (GTX109639) were purchased from GeneTex, anti-GAPDH (2118) were obtained from Cell Signaling Technology (Danvers, MA, United States). All the eukaryotic expression plasmids, including p3Xflag-*UL5*, pCMV-HA-*UL8*, pEGFPC1-*UL52*, were generated in our laboratory. Additional information about plasmids construction and primer sequences was shown in Supplementary Table 1. All siRNAs were purchased from Gene Pharma (Shanghai, China), and their sequences were shown in Supplementary Table 2.

### Cytotoxicity and Antiviral Activity Assay

For cytotoxicity assay, Vero cells were cultured in a 96-well plate (1 × 10^4^ cells/well) before the cells were incubated with different concentrations of Oleanolic acid or ACV for another 72 h. Cell viability was evaluated using a CCK8-kit. 5 μl of CCK8 was added to each well and the cells were incubated at 37°C for 1-2 h. The OD value at 490 nm was detected by a microplate reader.

To evaluate the antiviral activity, plaque reduction assay was performed. Briefly, Vero cells in 24-well plate were infected with HSV-1 (MOI = 1) at 37°C for 2 h to facilitate the absorption of viral particles. The culture medium was then replaced with maintenance medium containing 1% methylcellulose (SIJIA BIOTECH, Guangzhou, China) in the presence or absence of inhibitors. After 72 h incubation, the cells were fixed with 10% formalin and stained with 1% crystal violet (Beyotime, Suzhou, China). Total number of plaques was counted and the inhibitory ratio was calculated. In addition, viral titration was used to determine cytopathic effects (CPEs) in Vero cells. The 50% tissue culture infectious dose (TCID_50_) was calculated and converted into plaque-forming units (PFU)/ml. Cell lysates were diluted into different concentration gradients and were added into the Vero cells in 96-well plate. After 48 h incubation, the cellular CPEs were recorded and the titer TCID_50_ was calculated.

### Quantitative Real-Time PCR (qRT-PCR)

Total RNA was extracted with TRIZOL reagent (TIANGEN, Beijing, China) according to the manufacturer’s protocol. RNA concentration was measured using the P330 Nanophotometer (IMPLEN, Munich, Germany), and 1μg RNA was reverse-transcribed into cDNA using the PrimeScript RT Reagent Kit (TAKARA, Dalian, China). The mRNA expression of viral genes was analyzed by a Bio-Rad CFX96 Real-time PCR System (Bio-Rad) (Xiang et al., 2012). GAPDH was used as an internal reference. All primer information was shown in Supplementary Table 3.

To determine the copy number of viral genome DNA, HaCaT cells were infected with HSV-1 (MOI = 1) at 37°C for 2 h. Oleanolic acid (20 μM) was then added and incubated for another 24 h. The supernatant and cell pellets were collected, frozen at −80°C and thawed for three times. Virus genomic DNA was isolated using a UNIQ-10 viral DNA extraction kit (Sangon, China), and the DNA copy number of viral genes were determined using real-time DNA PCR.

### Viral Inactivation, Attachment, and Penetration Assay

For viral inactivation assay, HSV-1 virions (50 PFU/well) were incubated with Oleanolic acid (20 μM) at 37°C for 2 h. After incubation, the drug-virus mixture was added to Vero cells in 24-well plate and was adsorbed at 37°C for 2 h. The mixture was then removed and the cells were incubated with complete covering solution for 72 h to perform plaque assay. For viral attachment assay, pre-cooled Vero cells were incubated with HSV-1 (50 PFUs/well) and different concentrations of Oleanolic acid at 4°C for 2 h to allow the attachment of viral particles. The virus inoculum was then removed and the cells were washed and replenished with a cover layer. After 72 h incubation at 37°C, the cells were fixed and stained, the number of plaques was counted. For viral penetration assay, pre-cooled Vero cells were infected with HSV-1 at 4°C for 2 h. The culture medium was then replaced with medium containing Oleanolic acid, and the cells were incubated at 37°C for 10 min. After incubation, PBS buffer (pH = 3) was added to wash the cells three times, and alkaline PBS (pH = 11.0) was added for 1 min to remove the bound but not entered virions. Then the cells were replenished with a cover layer and incubated at at 37°C for 72 h to perform plaque assay.

### Infection Assay of EGFP-Tagged HSV-1

HaCaT cells cultured in a confocal dish (8 × 10^4^ cells/well) were infected with EGFP-HSV-1 (MOI = 1) in the presence or absence of Oleanolic acid (20 μM) for 2 h at 37°C. The cells were then fixed and stained with DAPI (Biotium). Fluorescent images were captured by a laser scanning microscope (Zeiss). Fluorescent intensity was calculated by Image J software.

### Western Blot Assay

Cells were lysed with sodium dodecyl sulfate (SDS) buffer (Beyotime, Shanghai, China) containing 1 mM phenylmethylsulfonyl fluoride (PMSF), and total proteins were separated by 8–15% gradient SDS-PAGE. The samples were then transferred to polyvinylidene fluoride (PVDF) membrane (Millipore), blocked with 5% non-fat milk at room temperature for 1 h, and incubated with different primary antibodies overnight at 4°C. Finally, the samples were incubated with appropriate secondary antibodies at room temperature for 1 h. Target proteins were detected by ECL solutions. The band intensity of each protein was calculated using Quantity One software (Bio-Rad, Hercules, CA, United States) and was normalized to GAPDH.

### Transfection of siRNA or Plasmid

HaCaT cells cultured in a 12-well plate (1.5 × 10^5^ cells/well) were infected with HSV-1 (MOI = 1) for 4 h and were then transfected with siRNA (100 nM) through Lipofectamine 3,000 transfection reagent. After 24 h, the sample was frozen and thawed three times, and virus titer was determined. For plasmid transfection, HaCaT cells were transfected with p3Xflag-*UL5*, pCMV-HA-*UL8*, pEGFPC1-*UL52* (2 μg) using Lipofectamine 3,000 transfection reagent. After 4 h, the culture media was changed to maintenance solution containing HSV-1 (MOI = 1) and Oleanolic acid (20 μM), and was incubated at 37°C for 24 h to detect virus titers.

### Co-immunoprecipitation (co-IP) Assay

Co-IP assay was performed as before (Wang et al., 2018). Briefly, HaCaT cells were transfected with plasmids overexpressing *UL5/UL8/UL52* before the cells were infected with HSV-1 (MOI = 5) in the presence or absence of Oleanolic acid (20 μM). After 2 h incubation, the medium was removed and replaced with fresh solution containing Oleanolic acid (20 μM) for another 2 h. Total cell lysates were then collected and lysed with ice-cold lysis buffer containing 1 mM PMSF. After determination of protein concentration using a BCA protein assay kit, 1 mg of protein was incubated with the indicated primary antibody or IgG at 1 μg for 4 h at 4°C before being agitated with 40 μl PLUS-Agarose (Santa Cruz Biotechnology) at 4°C overnight. The immunoprecipitated proteins were further collected by centrifugation (14,000 × *g*, 15 s) at 4°C. The unbound proteins were removed by washing five times with precooled lysis buffer containing 1 mM PMSF. Immunoprecipitated proteins were separated by adding 40 μl SDS loading buffer and boiling for 5 min and were then subjected to western blot analysis with specific primary and secondary antibodies.

### HSV-1-Infected Zosteriform Model

The HSV-1-infected zosteriform model was established as previous reports (van Lint et al., 2004; Wang et al., 2017, 2018). Briefly, 6–8 weeks aged female C57BL/6 mice were purchased from Guangdong Medical Experimental Animal Center (China). The hair of mice from spine to middle right part of abdomen was removed. After anesthesia, the exposed part was treated with 20-μl volume droplet containing 1 × 10^6^ PFU HSV-1. The skin was then scratched 20 times with a 27-gauge needle to allow the thoroughly absorption of viral droplet. After 24 h, the HSV-1-infected sites of mice were treated daily with 50 μl high-dose (1 mg/g) and low-dose (0.5 mg/g) Oleanolic acid gel preparation, or 50 μl ACV gel preparation (3 mg/g), respectively. The non-treated animals received the gel-only formulation without active ingredient. After continuous 9 days treatment, the mice were sacrificed, and the relative sizes of skin lesions were quantified by measuring the widths of the zosteriform lesions, which were used as an indicator of the severity of the skin lesion. Besides, the skin tissue, heart, liver, spleen, lung, and kidney were storage at −80°C for further western blot and qRT-PCR analysis.

### Statistical Analysis

Data were presented as mean ± SD of the results from at least 2 independent experiments. Student’s *t*-test was used to compare the two groups, with significance set as *P* < 0.05 (^∗^), *P* < 0.01 (^∗∗^), or *P* < 0.001 (^∗∗∗^). All the statistical analyses were correlated by SPSS10.0 statistical software and figures were draw by GraphPad Prism 7 software.

## Results

### Cytotoxicity and Antiviral Activity of Oleanolic Acid

Firstly, the cytotoxicity of Oleanolic acid on epithelial cells (Vero and HaCaT cells), or neuronal cells (SH-SY5Y cells) was detected by CCK8 assay (Supplementary Figures 1A–C). Accordingly, a non-cytotoxic concentration of Oleanolic acid was used to determine its antiviral activity. Viral plaque assay showed that Oleanolic acid significantly inhibited the plaque formation of HSV-1 F strain on Vero cells in a concentration-dependent manner (Figures 1B–D). In addition, Oleanolic acid (20 or 10 μM) significantly reduced HSV-1 DNA copy numbers (Figure 1E), virus titers (Figure 1F), and inhibited the protein levels of viral immediate-early protein ICP0, late protein gB and gD (Figure 1G). Furthermore, the anti-HSV-1 activity of Oleanolic acid on neuronal cells (SH-SY5Y and BV2 cells) was also examined. SH-SY5Y cells are neuroblastoma cells which are extensively used as neuron cell model to investigate the molecular mechanism of HSV-1 neuronal infection. BV2 cells are microglia cells exerting anti-HSV-1 immune activity in brain and are used to explore the interaction between HSV-1 infection and neuronal inflammation. Consistently, Oleanolic acid reduced the DNA copy number of different viral genes (Figure 1H), and virus titers (Figure 1I) at lower concentrations (5 and 2.5 μM) in both cells. Together, these results showed that Oleanolic acid had potent anti-HSV-1 activity in different cell models.

### Oleanolic Acid Inhibits ACV-Resistant HSV-1 Strains Infection

**FIGURE 2:**
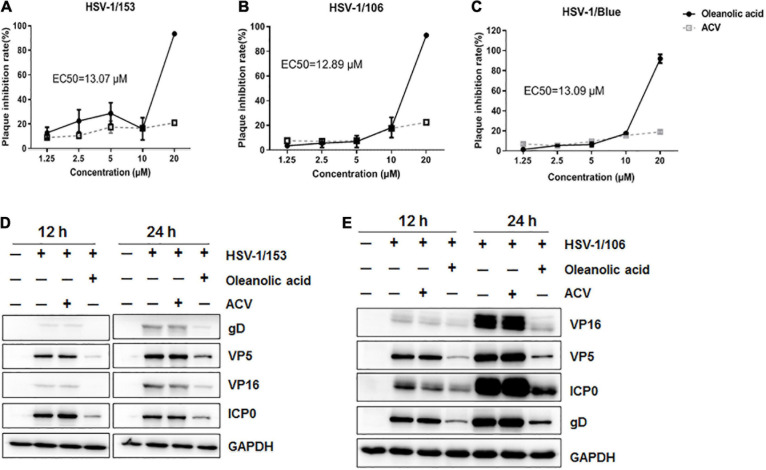
Oleanolic acid inhibits ACV-resistant strains. **(A–C)** Vero cells were infected with ACV-resistant strains HSV-1/153, HSV-1/106 and HSV-1/Blue (MOI = 1) in the presence of Oleanolic acid or ACV for 72 h, respectively. Plaque assay was performed and the plaque inhibition rate and EC_50_ value were calculated. Data are mean ± SD (*n* = 2). **(D,E)** HaCaT cells were treated with HSV-1/153 and HSV-1/106 (MOI = 1) in the presence of Oleanolic acid (20 μM) or ACV (20 μM) for 12 and 24 h, respectively. Cell lysates were then subjected to western blot assay to detect the protein levels of viral protein ICP0, VP5, VP16, and gD. GAPDH was used as a loading control.

Acyclovir exerts its antiviral activity mainly through inhibiting HSV Thymidine kinase (TK) to interrupt viral DNA replication, and clinical isolated ACV-resistant HSV strains were mostly TK-negative, TK-low-producer mutants and TK-altered mutants (Burns et al., 1982). Herein, we assessed whether Oleanolic acid had antiviral activity against ACV-resistant strains, including HSV-1/Blue, a TK mutant derived from HSV-1 KOS strain, and two clinic isolated ACV-resistant HSV-1 strains, HSV-1/106 and HSV-1/153 (Jin et al., 2014). HSV-1 F and KOS strain were originally isolated from facial lesions and exhibited lower mortality in comparison to clinical HSV-1strains (Dix et al., 1983). Besides, F and KOS strain demonstrated different transcriptional changes and viral gene or protein expression patterns in infected human neuronal cells, whereas no difference in infected epithelial cells (Mangold et al., 2021). Accordingly, we examined the antiviral activity against ACV-resistant HSV-1 strains in epithelial Vero cells. As shown in Figures 2A–C, the plaque inhibitory rates of ACV toward HSV-1/Blue, HSV-1/106, and HSV-1/153 were less than 20% even at a high concentration (20 μM). On the contrary, Oleanolic acid (20 μM) exhibited a nearly 100% inhibitory rate against HSV-1/Blue, HSV-1/106 and HSV-1/153, respectively. The EC_50_ values were shown in Table 1. In addition, western blot assay demonstrated that Oleanolic acid significantly reduced the protein levels of viral protein VP5, VP16, gD, and ICP0 of ACV-resistant strains, while ACV treatment had no effect (Figures 2D,E). Therefore, Oleanolic acid can inhibit ACV-resistant HSV-1 strains.

**TABLE 1:** Cytotoxic effect and antiviral activity of ACV and Oleanolic acid.

**Drug**	**IC_50_ (μM)**			**EC_50_ (μM)**			
	**Vero**	**SH-SY5Y**	**HaCaT**	**HSV-1/F**	**HSV-1/106**	**HSV-1/153**	**HSV-1/blue**
ACV	>50	>50	>50	<0.5625	>20	>20	>20
Oleanolic acid	39.05 ± 0.561	20.5 ± 0.325	37.06 ± 0.401	4.712 ± 0.321	12.89 ± 0.681	13.06 ± 0.512	13.09 ± 0.642

### Oleanolic Acid Inhibits HSV-1 Early Infection

**FIGURE 3:**
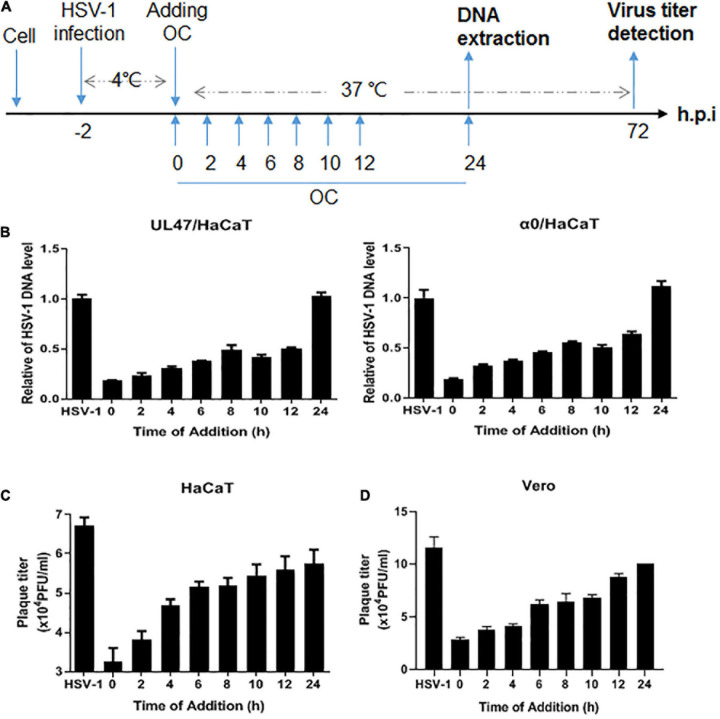
Oleanolic acid affects viral early infection. **(A)** Simple diagram of the time-of-addition assay. **(B)** HaCaT cells infected with HSV-1 (MOI = 1) at 4°C for 2 h were washed with PBS and incubated at 37°C. Oleanolic acid were added at indicated time points. After 24 h, total viral DNA was extracted and the DNA copy numbers of viral gene *α0* and *UL47* were detected by qRT-PCR. **(C,D)** HaCaT and Vero cells were infected with HSV-1 (MOI = 1) at 4°C for 2 h, and were washed with PBS to remove the virions. The cells were then incubated at 37°C and were treated with Oleanolic acid at indicated time points. After 72 h, the supernatant of cell culture was collected to perform viral titration assay and calculate viral titers. Data are mean ± SD (*n* = 3).

To determine the specific stage that Oleanolic acid inhibited HSV-1 infection, we performed a time-of-addition assay (Figure 3A). As shown in Figures 3B,C, Oleanolic acid exhibited the highest inhibitory effects on viral genomic DNA copy numbers and virus titers at 0–4 h in HaCaT cells. Similarly, viral titers were also significantly reduced when Oleanolic acid was added at 0–4 h in Vero cells (Figure 3D). These results clearly suggested that Oleanolic acid mainly suppressed HSV-1 early infection. We then examined whether Oleanolic acid affected the expression of viral immediate-early gene and found that Oleanolic acid did not affect the mRNA expression of viral immediate early gene (Supplementary Figures 2A–D) and protein levels (Supplementary Figure 2E). Oleanolic acid also did not affect the production of interferons (Supplementary Figures 3A–C). In addition, we examined several events occurring in HSV-1 early infection stage, including viral inactivation, attachment and penetration, and found that Oleanolic acid neither directly inactivated virus particles (Supplementary Figure 4A), nor affected viral attachment and penetration (Supplementary Figures 4B,C).

### Oleanolic Acid Inhibits Viral Helicase-Primase Complex

**FIGURE 4:**
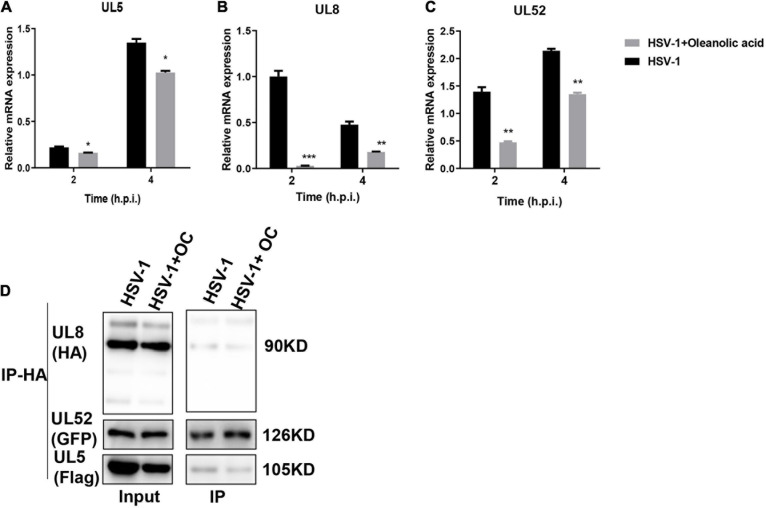
Oleanolic acid inhibits the expression of genes related to the HSV-1 helicase-primase complex. **(A–C)** HaCaT cells were treated with HSV-1 (MOI = 5) and Oleanolic acid (20 μM) for 2 and 4 h. Total RNA was extracted and the mRNA expression levels of gene *UL5*, *UL8*, and *UL52* were detected by RT-qPCR. Data are mean ± SD (*n* = 3). *p < 0.5, **p < 0.01, ***p < 0.001. **(D)** The cells were transfected with overexpression plasmid Flag-*UL5*, HA*-UL8*, and EGFP-*UL52* for 24 h and were infected with HSV-1 (MOI = 5) in the presence or absence of Oleanolic acid (20 μM) for 4 h. Cell lysates were collected and subjected to immunoprecipitation with anti-HA antibody and protein A/G-agarose. Immunoprecipitates were detected by western blot assay with anti-GFP, anti-HA and anti-Flag antibody.

Considering the different antiviral actions between Oleanolic acid and ACV, we speculated that Oleanolic acid might affect the viral helicase-primase complex. Indeed, the mRNA expression levels of HSV-1 helicase-primase complex, including *UL5*, *U52*, and *UL8*, were significantly downregulated by Oleanolic acid (Figures 4A–C), suggesting that Oleanolic acid might inhibit HSV-1 early infection by affecting the helicase-primase complex. Among these genes, Oleanolic acid showed the highest inhibitory effect on *UL8* (Figure 4B). Next, we explored whether Oleanolic acid affected the interaction among UL5, UL8, and UL52. Due to the lack of commercial antibodies of HSV-1 UL5, UL8, and UL52, we constructed Flag-tagged *UL5*, HA-tagged *UL8*, and EGFP-tagged *UL52* plasmid, respectively. HaCaT cells were transfected with these plasmids and were challenged with HSV-1 and Oleanolic acid. Unexpectedly, the co-immunoprecipitation assay showed that Oleanolic acid did not affect the interaction between UL8 and UL52 or between UL8 and UL5 (Figure 4D), implying that Oleanolic acid did not affect the formation of helicase-primase complex.

### *UL8* Is Involved in the Antiviral Activity of Oleanolic Acid

**FIGURE 5:**
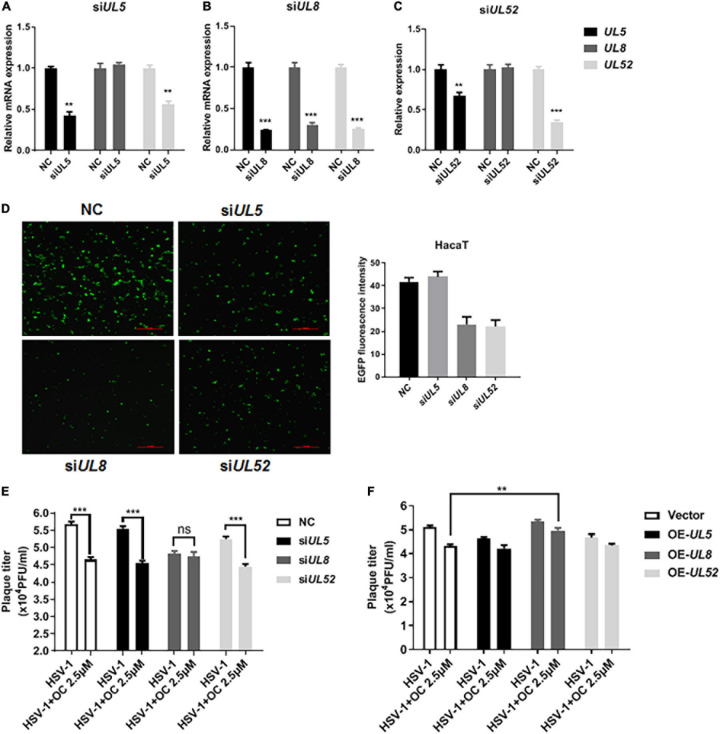
*UL8* is involved in the antiviral action of Oleanolic acid. **(A–C)** HaCaT cells were firstly infected with HSV-1 (MOI = 1) for 2 h and were then transfected with siRNAs targeting *UL5*, *UL8*, and *UL52*, respectively. After 24 h incubation, total RNA was extracted and the mRNA expressions of *UL5*, *UL8*, and *UL52* were detected by qRT-PCR. **(D)** HaCaT cells were infected with the EGFP-HSV-1 strain in the presence of different siRNAs for 24 h. The cells were then fixed and immunofluorescence images were taken under a fluorescence microscope. The fluorescence intensity was determined by ImageJ. **(E)** HaCaT cells were firstly infected with HSV-1 (MOI = 1) for 2 h and were then transfected with siRNAs for 24 h. The cells were then treated with Oleanolic acid (20 μM) for another 24 h. The supernatant and cell pellets were frozen at −80°C and thawed three times, diluted into different concentration gradients and added into the Vero cells in 96-well plate. The CPE effect was observed and the viral titer TCID_50_ was calculated. **(F)** HaCaT cells were transfected with overexpression-plasmid *UL5*, *UL8*, and *UL52* at 37°C for 24 h, respectively. The cells were then challenged with HSV-1 (MOI = 1) and Oleanolic acid (20 μM) for another 24 h. The supernatant and cell pellets were collected and viral titer was calculated. Data are mean ± SD (*n* = 3). **p < 0.01, ***p < 0.001. ns, not significant.

Next, we analyzed the possible cross-impacts among *UL5*, *UL8*, and *UL52* using specific siRNAs. As shown in Figures 5A–C, all siRNAs effective downregulated their corresponding mRNA expression levels. Interestingly, knockdown of *UL5* or *UL52* reduced the mRNA expression of both *UL5* and *UL52*, without effect on *UL8*. By contrast, knockdown of *UL8* significantly reduced all three genes, implying the central role of *UL8* in gene expression. In addition, HaCaT cells were infected with EGFP-tagged HSV-1, and only *UL8*- or *UL52*-targeted siRNA reduced the fluorescent intensity of EGFP (Figure 5D), indicating that *UL8* and *UL52* were critical for HSV-1 life cycle. Considering the reductive effects of Oleanolic acid on *UL5*, *UL8*, and *UL52* (Figures 4A–C), it was reasonable that Oleanolic acid exerted its antiviral activity via deregulating *UL8*, which in turn, downregulated *UL52* and *UL5* expression, resulting in reduced viral replication.

Furthermore, we examined the effects of siRNA-mediated knockdown or plasmid-mediated overexpression of *UL5*, *UL8*, and *UL52* on the antiviral activity of Oleanolic acid, respectively. HaCaT cells were transfected with siRNAs or plasmids before HSV-1 infection in the presence of Oleanolic acid for 24 h, and virus titers were analyzed. As shown in Figure 5E, knockdown of *UL5* and *UL52* did not affect the antiviral activity of Oleanolic acid, whereas no significant difference was observed between the Oleanolic acid and virus control groups in the presence of siRNA-*UL8.* Consistently, overexpression of *UL8* restored virus titers reduced by Oleanolic acid, while overexpression of *UL5* and *UL52* had no effects (Figure 5F). These results clearly suggested the involvement of UL8 deregulation in Oleanolic acid-mediated antiviral activity. Furthermore, we performed molecular docking to investigate whether Oleanolic acid may directly interact with viral protein UL8/UL5/UL52, as well as other HSV-1 early protein (e.g., ICP0, ICP4, and ICP27), to affect viral early infection. However, we found that there were no direct interactions between Oleanolic acid and these viral proteins. Accordingly, we postulated that Oleanolic acid might affect the protein stability of other cellular components critical for ICP0-mediated immediate early gene expression and/or UL8-mediated viral gene replication. Together, these above results indicated that Oleanolic acid inhibited HSV-1 infection partly via deregulating UL8, and further works are required to investigate the molecular mechanism by which Oleanolic acid inhibits UL8.

### Oleanolic Acid Ameliorates HSV-1 Infection-Induced Skin Lesions

**FIGURE 6:**
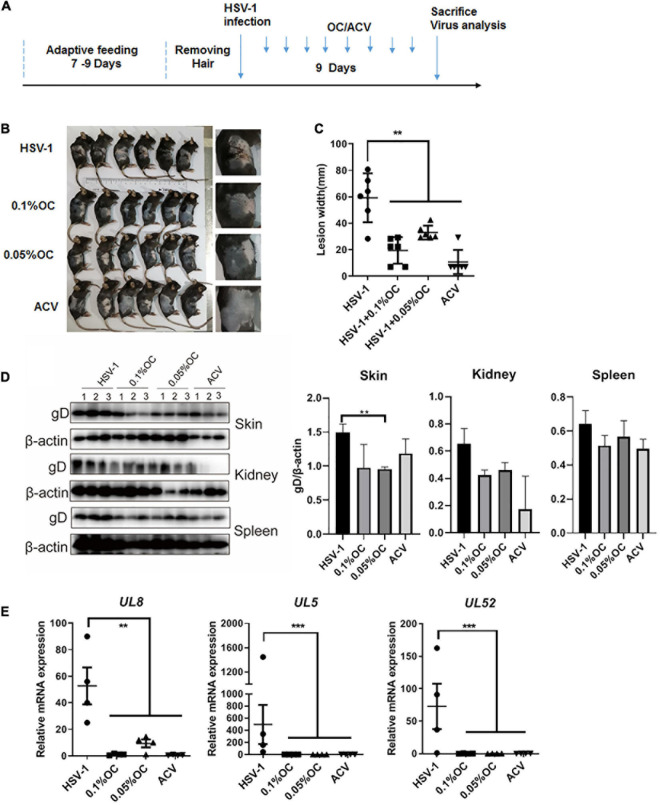
Oleanolic acid reduces HSV-1 infection-induced skin lesions in mice. **(A)** Simple diagram of the *in vivo* experiment. **(B)** Gel preparations containing high or low concentration of Oleanolic acid (OC) were applied to mice with zosteriform model of HSV-1 infection once a day. After 9 consecutive days, the mice were then sacrificed for photographing. **(C)** The widths of the zosteriform lesions were counted by ImageJ software. **(D)** Total proteins in skin, kidney, and spleen were extracted, and the protein levels of viral protein gD were detected by western blot assay. Protein band intensity was quantitative analyzed by Image J software. **(E)** Viral RNA in HSV-1-infected skin tissues was extracted, and the mRNA expression levels of *UL5*, *UL8*, and *UL52* were detected by RT-qPCR. Data are mean ± SD (*n* = 9). **p < 0.01, ***p < 0.001.

Finally, we utilized the skin herpes model, a classic HSV-1 infection animal model, to confirm the antiviral activity of Oleanolic acid (van Lint et al., 2004; Wang et al., 2016) (Figure 6A). Gel mixture containing 0.05 and 0.1% Oleanolic acid were used to treat abraded skin once per day starting 24 h after the HSV-1 infection-induced skin lesions had significantly manifested. Apparently, compared to control mice treated with gels lacking drug, Oleanolic acid and ACV effectively ameliorated the skin lesions and reduced the width of the zosteriform lesions caused by HSV-1 infection (Figures 6B,C). Western blot assay also clearly indicated that the protein levels of viral protein gD in skin and kidney were downregulated by Oleanolic acid and ACV, whereas no difference was observed in spleen (Figure 6D). Importantly, Oleanolic acid exhibited the strongest inhibitory effect on the protein level of gD in skin, even higher than ACV, confirming the antiviral activity of Oleanolic acid. In addition, the mRNA expression levels of viral genes in skin, including *UL5*, *UL8*, and *UL52*, were significantly reduced by Oleanolic acid (Figure 6E). Interestingly, the reduction of gD level by Oleanolic acid was less than the extremely low level of *UL5*, *UL8*, and *UL52* transcripts. One possible explanation was that gD is a late glycoprotein on viral outer-membrane that does not require the large *de novo* synthesis, which might be affected by UL8-mediated gene replication. Together, these results indicated that Oleanolic acid gels reduced the expression levels of the helicase-primase genes, which might contribute to suppress the progression of zosteriform disease *in vivo*.

## Discussion

Natural products are important antiviral drug resources, and increasing evidence has proved the anti-HSV-1 activities of crude extracts and pure compounds isolated from plants. For instance, natural extracts containing phenols, flavonoids, alkaloids, saponins, steroids, glycosides, and tannins, have been found to interfere with the replication process of HSV-1 infection (Khan et al., 2005; Schnitzler et al., 2008; Son et al., 2013; Hassan et al., 2015). Our study also showed that Oleanolic acid, a triterpenoid isolated and purified from benzoin, has antiviral activity against both normal HSV-1 and ACV-resistant strains, including HSV-1/blue, HSV-1/106 and HSV-1/153 (Figure 2). Although Oleanolic acid has been shown to inhibit the infection of various viruses, such as HIV, influenza virus and hepatitis virus (Zhu et al., 2001; Ikeda et al., 2005; Yu et al., 2006; Zhao et al., 2012; Kong et al., 2013; Wang et al., 2016; Yang et al., 2018), the detailed mechanism about how Oleanolic acid inhibits herpes and influenza viruses remain unclear. Herein, we demonstrated that Oleanolic acid might exert its anti-HSV-1 activity through deregulating *UL8*, one component of the helicase-primase complex. We also confirmed its anti-HSV-1 activity *in vivo* using skin herpes model.

Through a time-of-addition assay, we found that Oleanolic acid mainly inhibited HSV-1 early infection (Figure 3). However, Oleanolic acid neither affected viral adsorption and penetration, nor directly inactivated virus particles (Supplementary Figure 4). Besides, the mRNA and protein levels of viral immediate early genes were also not influenced by Oleanolic acid treatment (Supplementary Figure 2). Considering that Oleanolic acid had potent antiviral activity against ACV-resistant strains, we next tested the possible impact of Oleanolic acid on viral helicase-primase complex, a potential therapeutic target of HSV-1 infection (James and Prichard, 2014). Indeed, there were several helicase-primase inhibitors (HPIs) (e.g., pritelivir and Amenamevir) that have been reported to stabilize the binding capacity of the helicase-primase complex and thereby inhibited HSV-1 replication (James and Prichard, 2014). Our study showed that Oleanolic acid could significantly down-regulate the mRNA expression of *UL5*, *UL8*, and *UL52* in the immediate early stage (Figures 4A–C), without effect on their interactions between UL8 and UL5 or UL52 (Figure 4D). Besides, knockdown of *UL8* or *UL52*, but not *UL5*, inhibited HSV-1 infection (Figure 5D). *UL5* and *UL52* exhibit a complex interdependence, and *UL5*/*UL52* is co-translated to generate a functional complex. For instance, without UL5, UL52 is insoluble, whereas UL5 is inactive without UL52. Consistently, we showed that knockdown of *UL5* or *UL52*, respectively, could reduce the mRNA expression of both *UL5* and *UL52* (Figures 5A–C). The UL8 subunit regulated the nucleation of UL5/UL52 and cooperated with other components of the HSV replication machinery, including UL9, ICP8, and UL30 DNA Pol (Muylaert et al., 2011), suggesting that UL8 might play a critical role in integrating activity at the replication fork, and interruption of UL8 represents a promising antiviral strategy. More importantly, we found that knockdown of *UL8* significantly affected the antiviral activity of Oleanolic acid (Figure 5E). In addition, only overexpressing *UL8* could restore the decrease of virus titers caused by Oleanolic acid (Figure 5F). Therefore, we concluded that Oleanolic acid exerted its anti-HSV-1 activity through deregulating *UL8*, and subsequently *UL5* and *UL52*. We further performed molecular docking assay and fail to find the possible direct interaction between UL8 and Oleanolic acid. One possible explanation was that Oleanolic acid affected the protein stability of other cellular components critical for UL8 and UL8-mediated viral gene expression and further works are required to investigate the interaction between UL8 and Oleanolic acid extensively. Finally, we demonstrated that topical application of gels containing 0.05 or 0.1% Oleanolic acid remarkably improved skin zosteriforms caused by HSV-1 infection (Figure 6), suggesting the interruption of UL8-mediated helicase-primase complex as promising therapeutic targets for HSV-1-related skin lesions, such as *H. gladiatorum*. Considering that Oleanolic acid inhibited HSV-1 infection in neuronal cells (Figures 1G,H), it is interestingly to examine whether Oleanolic acid can inhibit herpes simplex encephalitis *in vivo*, another important diseases caused by HSV-1 neuronal infection. It is also unclear whether the same effective concentrations of Oleanolic acid can be achieved after systemic administration to against HSV-1 neuronal infection. Moreover, we cannot exclude the possibility that except for UL8, there were other viral or cellular targets involved in the anti-HSV-1 mechanism of Oleanolic acid *in vivo*.

In summary, our works demonstrated that Oleanolic acid could inhibit ACV-sensitive and ACV-resistant HSV-1 strains through influencing the helicase-primase complex, especially involving the UL8 subunit. By deregulating UL8, Oleanolic acid might be used alone or in combination to treat HSV-1-induced skin lesions, especially in ACV-resistant populations.

## Data Availability Statement

The raw data supporting the conclusions of this article will be made available by the authors, without undue reservation.

## Ethics Statement

The animal study was reviewed and approved by Medical Ethics Committee of Jinan University.

## Author Contributions

TS, JY, and JJ conceived and designed the study. TS wrote the first draft of the manuscript. TS, JJ, ZW, and YJ developed or designed the methodology and specifically performed the experiments. JY and YLW provided study materials. KZ commented or revised the manuscript, including pre-or postpublication stages. YFW and ZR were responsible for management and coordination for the research activity planning and execution and acquisition of the financial support for the project leading to this publication. All authors contributed to the article and approved the submitted version.

## Conflict of Interest

The authors declare that the research was conducted in the absence of any commercial or financial relationships that could be construed as a potential conflict of interest. The reviewer QZ declared a shared affiliation with one of the authors, KZ, to the handling editor at the time of review.
