# Supplementary material for: Viral UL8 Is Involved in the Antiviral Activity of Oleanolic Acid Against HSV-1 Infection
Source: Front Microbiol. 2021 Jul 20;12:689607. doi: 10.3389/fmicb.2021.689607 (PMC8329587; doi:10.3389/fmicb.2021.689607)
Supplement: Supplementary file 1 [file Data_Sheet_1.PDF]

Supplemental information

Viral UL8 is involved in the antiviral activity of Oleanolic acid against HSV-1 infection

Supplemental Figures and Legends

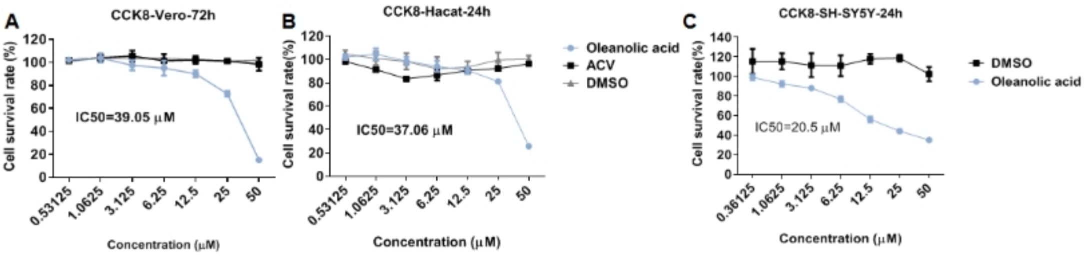

Figure S1: The cellular toxicity of Oleanolic acid. (A-C) Treatment of Oleanolic Acid at different concentrations on Vero cells for 72 h, HaCaT cells for 24 h and SH-SY5Y cells for 24 h, respectively. The absorbance was then detected by CCK8 assay and cell survival rate was calculated according to the absorbance.

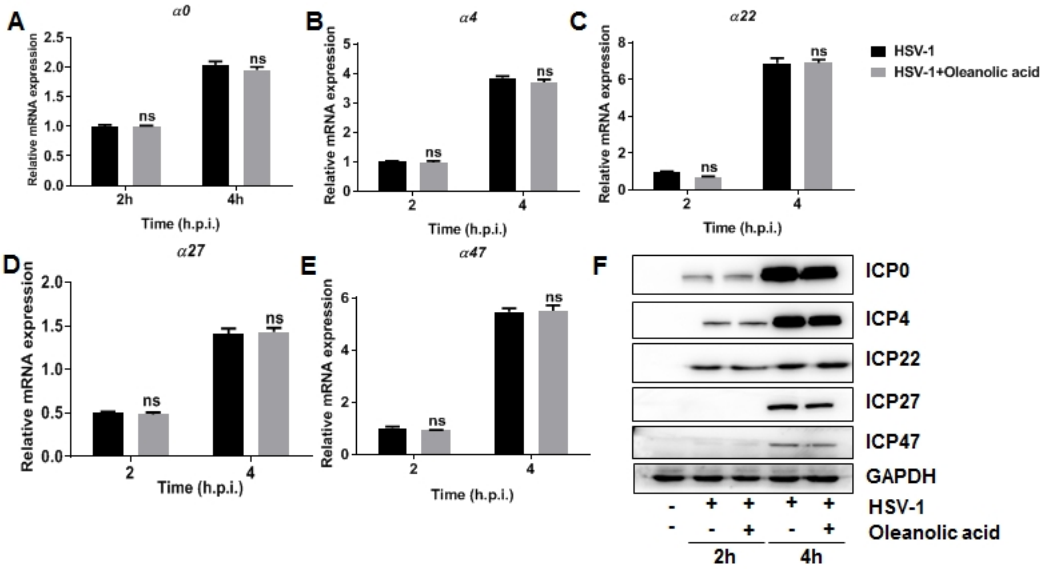

Figure S2: The effect of Oleanolic acid on the immediate early genes and proteins of HSV-1. (A-E) HaCaT cells were infected with HSV-1 (MOI=5) in the presence or absence of Oleanolic Acid (20 μM) for 2 h and 4 h. The total RNA was then extracted and reversed into cDNA, and the mRNA expression levels of HSV-1 early genes were detected by RT-qPCR. (F) HaCaT cells were treated with HSV-1 (MOI=5) and Oleanolic Acid for 2 h and 4 h, respectively. Total proteins were collected for western-blot detection of early protein expression.

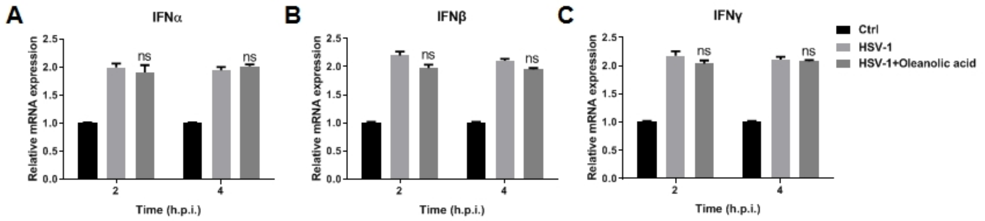

Figure S3: Oleanolic acid does not affect the expression of interferons. (A-C) HaCaT cells were treated with HSV-1 (MOI=5) and Oleanolic Acid (20 μM) for 2 h and 4 h. The sample RNA was then extracted and the expression levels of *IFN-α*, *IFN-β*, *IFN-γ* were detected by RT-qPCR.

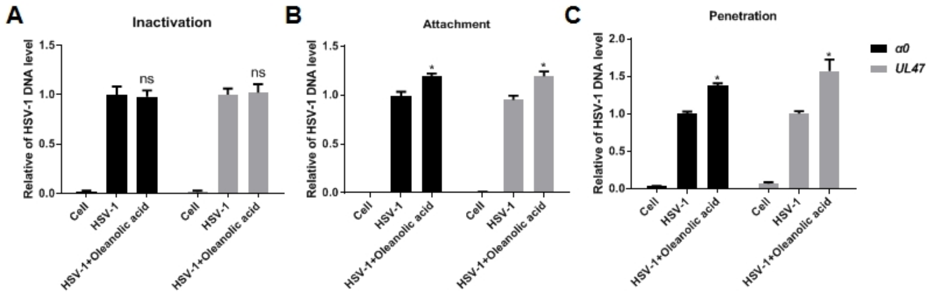

Figure S4: Effect of Oleanolic acid on viral direct inactivation, attachment and penetration. (A) HSV-1 viral particles (MOI=1) were incubated with Oleanolic Acid (20 μM) for 2 h, and were then applied to HaCaT cells for 24 h to detect the DNA copy number of virus genome. (B) HaCaT cells were treated with HSV-1 (MOI=10) and Oleanolic Acid at 4 °C for 2 h, washed with PBS for three times and cultured for 24 h, which was followed by detection of virus genomic DNA copy number. (C) HaCaT cells were treated with HSV-1 (MOI=10) and Oleanolic Acid at 4 °C for 2 h, and then transferred to an incubator for 15 min. The cells were then washed with PBS for another 24 h, and the DNA copy number of the virus genome was detected.

Supplemental Tables

Supplemental Tables 1: List of primers used for plasmids construction

Plasmids Name	Primer Sequence
HA-*UL8-EcoR Ⅰ*-F	CGGAATTCGGATGGACACCGCAGATATCGT
HA-*UL8-Kpn Ⅰ*-R	GGGGTACCATTTATTGGTCAAACTCAGGCA
EGFP-*UL52-HinDⅢ*-F	CCAAGCTTCTATGGGGCAGGAAGACGGG
EGFP-*UL52-BamH Ⅰ*-R	CGGGATCCTCAAGACGACGGTTGAGAGGTG
Flag-*UL5*-1-F	ACGATGACAAGCTTGCGGCCATGGCGGCGGCCGGCGGG
Flag-*UL5*-1-R	TCTGGCTGTTGAGGACGTAAGTGACGTCGTTGC
Flag-*UL5*-2-F	TTACGTCCTCAACAGCCAGATCGCGGTAAC
Flag-UL5-2-R	AGGGATGCCACCCGGGATCCTTAATATACAATGACCACGTTCGGA

Supplemental Tables 2: Sequence of siRNAs

Target Name	siRNA Sequence
si*UL5*-1（Human）	Sense: GCAGCAACGTGATCGTCAT
	Antisense: ATGACGATCACGTTGCTGC
si*UL5*-2（Human）	Sense: GGTTGTTCTCCTCCCACAA
	Antisense: TTGTGGGAGGAGAACAACC
si*UL8*-1 （Human）	Sense: GGGACTGGTGGTGAAAGTT
	Antisense: AACTTTCACCACCAGTCCC
si*UL8*-2（Human）	Sense: GCGCGAATACCAGACTCTT
	Antisense: AAGAGTCTGGTATTCGCGC
si*UL52*-1（Human）	Sense: GGAGCAAGACAGGTTCGAA
	Antisense: TTCGAACCTGTCTTGCTCC
si*UL52*-2（Human）	Sense: CCATGTTCGTCTGTCGCTT
	Antisense: AAGCGACAGACGAACATGG

Supplemental Tables 3: Primers used for qRT-PCR

Gene Name	Primer Sequence
α0-F	CCCACTATCAGGTACACCAGC
α0-R	CTGCGCTGCGACACCTTTT
α27-F	TGGCGGACATTAAGGACATTG
α27-R	TGGCCGTCAACTCGCAGA
UL47-F	TACGAGGAGGACGACTACCC
UL47-R	ATCCGGACACGGGTAAAACC
UL5-F	GCACGAGTTCGGTAACCTCA
UL5-R	ACTCCTTGACCGACACGAAC
UL8-F	TCCGGTGGTGATGTTAACGG
UL8-R	GCAGATATCGTGTGGGTGGA
UL29-F	CATGCCGGATTTTAGCCGTG
UL29-R	TCGTGGTTTTCGTCAAACGC
UL30-F	TAACTGTACGGCGGACAACC
UL30-R	CAGCTCGTTCAGGTGGGATT
UL52-F	AGGCCATCAAGGACATCTGC
UL52-R	AATACGGCGCTCCACGTAAA
IFN-α-F	CTCATACACCAGGTCACGCT
IFN-α-R	AGTGTAAAGGTGCACATGACG
IFN-β-F	ACTGGCTGGAATGAAACCGT
IFN-β-R	GGCACAGCTTCTGTACTCCT
IFN-γ-F	GCTACACACTGCATCTTGGC
IFN-γ-R	CATGTCACCATCCTTTTGCCAG
GAPDH(human)-F	CACCATCTTCCAGGAGCGAG
GAPDH(human)-R	AGAGGGGGCAGAGATGATGA
